# Comparison of Early Postoperative Outcomes for Vaginal Anterior Sacrospinous Ligament Fixation with or without Transvaginal Mesh Insertion

**DOI:** 10.3390/jcm12113667

**Published:** 2023-05-25

**Authors:** Louis-Edouard Galan, Stéphanie Bartolo, Céline De Graer, Sophie Delplanque, Marine Lallemant, Michel Cosson

**Affiliations:** 1 Pôle Femme Mère Nouveau-né, Centre Hospitalier Universitaire de Lille, University of Lille, Avenue Eugène Avinée, 59000 Lille, Francemichel.cosson@chu-lille.fr (M.C.); 2 Douai Hospital, Route de Cambrai-BP 10740 CEDEX, 59507 Douai, France; stephanie.bartolo@ch-douai.fr; 3 Department of Obstetrics and Gynecology, Hôpital Erasme, Université Libre de Bruxelles, 1070 Brussels, Belgium; 4 Pôle Mère-Femme, Service de Gynécologie-Obstétrique, Centre Hospitalier Universitaire Besançon, Université de Franche-Comté, 3 Boulevard Alexandre Fleming, 25000 Besançon, France; marine.lallemant@wanadoo.fr

**Keywords:** pelvic organ prolapse, apical prolapse, vaginal approach, anterior sacrospinous ligament fixation, native tissue repair, mesh repair, postoperative complication

## Abstract

Pelvic organ prolapse affects one in three women, and cystocele accounts for 80% of the indications for surgery. Following the withdrawal of transvaginal mesh from the market, the objective of the present before-and-after study was to compare of the previous reference technique (Uphold^TM^ (Boston Scientific, Marlborough, MA, USA) mesh insertion) with anterior sacrospinous ligament fixation with suturing in terms of the outcomes 2 months after surgery. We performed a retrospective, observational, before-and-after study at Lille University Medical Center (Lille, France) by including consecutive patients operated on between 2011 and 2018 for Uphold^TM^ mesh insertion and between 2018 to 2020 for anterior sacrospinous ligament fixation. The primary outcome was the early recurrence of prolapse, and the secondary outcomes were the occurrence of early per-operative or post-operative complications and the development of de novo stress urinary incontinence. Here, 466 patients were included in the study (382 in the Uphold^TM^ group and 84 in the anterior sacrospinous ligament fixation group). The failure rate at 2 months was 6.0% (5 out of 84) for anterior sacrospinous ligament fixation and 1.3% (5 out of 382) for Uphold^TM^ (*p* < 0.01). The prevalence of acute urinary retention was significantly lower in the anterior sacrospinous ligament fixation group (3.6%) than in the Uphold^TM^ group (14.1%; *p* < 0.01), as was the de novo stress urinary incontinence rate (11.9% vs. 33.8%, respectively; *p* < 0.01). Anterior sacrospinous ligament fixation appears to be an effective, safe alternative to mesh insertion in the management of cystocele via the vaginal approach; the early complication rate was slightly lower, but the early failure rate was slightly higher.

## 1. Introduction

Pelvic organ prolapse is a common functional pathology that affects up to half of parous women and one third of women overall [1]. The lifetime incidence of surgery for pelvic organ prolapse is 11–19% [2,3]. Many symptoms can be linked to the presence of a pelvic organ prolapse, such as chronic pelvic pain, urinary symptoms such as bladder overactivity, nocturia, or even digestive symptoms. For all these pelvic organ prolapse-related symptoms, leading scientific societies agree that only urinary incontinence is curable by surgery. Nevertheless, some experts support an integral theory involving an interconnection between all the pelvic structures and claim that an adapted correction of a prolapse could improve or cure some or all symptoms [4]. Although 85% of women are satisfied with the functional outcome, the re-operation rate is high (around 30%) [3].

Anterior compartment prolapse (also referred to as cystocele) is the main indication for surgery [5] and is associated with apical compartment prolapse in the majority of cases [6]. Techniques with laparoscopic or transvaginal approaches have been developed for the surgical management of cystocele. The vaginal approach has several advantages over laparoscopy: a shorter operating time, more rapid resumption of daily activities, and lower treatment costs [7]. However, the latest Cochrane meta-analysis [7] found that the prolapse recurrence rate was twice as high for transvaginal surgery with native tissue repair than for transvaginal surgery with polypropylene mesh. Furthermore, the re-operation rate for prolapse recurrence was higher for native tissue repair. However, the meta-analysis emphasized the lower likelihood of repeat surgery, due to the absence of exposure to the mesh prosthesis. In this context, meshes were withdrawn first from the American market and then (in April 2019) worldwide [8].

The remaining vaginal techniques are based on native tissue repair. Anterior colporrhaphy is the technique most frequently used to treat anterior prolapse, but the success rate is low (between 30 and 60%) [9]. In cases with apical prolapse, anterior colporrhaphy must be combined with posterior sacrospinous ligament fixation [10,11]. The latter technique does not enable good correction of the vaginal axis [12]; this problem prompted Winkler et al. to develop anterior sacrospinous ligament fixation (ASSLF) in 2001 [13]. ASSLF may reduce the risks of anterior prolapse recurrence and rectal injury [14]. In the absence of an indication such as symptomatic fibroma, ASSLF does not have to be performed concomitantly with hysterectomy; hence, treatment with ASSLF alone shortens the length of hospital stay and facilitates the outpatient management of prolapse.

The objective of the present study was to compare the anterior insertion of a transvaginal mesh (Uphold^TM^ (Boston Scientific, Marlborough, MA, USA)) with ASSLF in terms of early recurrence, perioperative complications, and post-operative complications at 2 months.

## 2. Materials and Methods

We performed a single-center, retrospective, observational, descriptive before-and-after study at Lille University Medical Center (Lille, France). Our center has significant expertise in prolapse surgery, and more than 3000 gynecological procedures are performed there each year. We included all consecutive patients having undergone ASSLF for isolated or predominant anterior prolapse between 1 May 2019 and 31 December 2020 (i.e., a 20-month period).

These results were compared with those published prior to the date of withdrawal of the Uphold^TM^ prostheses from the market; we had recorded all consecutive cases of anterior compartment surgery with transvaginal mesh performed at Lille University Medical Center between 2011 and 2018 [15].

All of the patients in the present study were operated on by the same surgical team (including residents, fellows, and hospitalists). All of the indications for vaginal surgery were set by the same senior resident, who applied the same criteria during the two study periods. Patients who were treated with a technique other than ASSLF or Uphold^TM^ transvaginal mesh insertion were excluded, as were those who were operated on by surgeons who might have changed their practice following the withdrawal of the mesh from the market.

All of the patients gave their written, informed consent for the use of their personal medical data for research purposes.

The study’s primary outcome was the early recurrence (within 2 months of surgery) of anterior or apical pelvic organ prolapse. The secondary outcomes were the occurrence of a peri or postoperative complication and the onset of de novo stress urinary incontinence (SUI) within 2 months of surgery.

We defined recurrence as composite criteria combining the reappearance of clinical symptoms of prolapse during the immediate postoperative period or the following 2 months, with the need for re-operation in the anterior and/or apical compartment [16].

Each patient’s medical history was documented by reviewing their medical records and surgical reports. Postoperative data were collected from the notes made during a standard, scheduled consultation 2 months after surgery.

The ASSLF technique consisted of infiltration of the anterior vaginal wall with 1% xylocaine in physiological saline solution, anterior colpotomy, bilateral dissection of the vesicovaginal space and the paravesical fossa, hooking of the sacrospinous ligament with the Capio^TM^ suture-capturing device (Boston Scientific, Natick, MA, USA), and fixation to the floor of the vagina. Vaginal suturing was initiated before tensioning of the nonabsorbable anchoring sutures and completed after tensioning. In some cases, two sets of anchoring sutures were placed through each sacrospinous ligament. The ASSLF procedure could be combined with additional procedures for the treatment of cystocele or rectocele with native tissue repair.

We performed a descriptive analysis of the study population and postoperative complications. Furthermore, we performed an explanatory univariate analysis by comparing percentages in a chi-squared test or Fisher’s exact test, depending on the sample size. Medians were compared in a nonparametric Kruskal−Wallis test. The threshold for statistical significance was set to *p* < 0.05. A multivariate logistic regression analysis was then performed on variables with *p* < 0.10 in the univariate analysis. All of the analyses were carried out using Stata software (version 13.0.0, StataCorp LP, College Station, TX, USA).

## 3. Results

### 3.1. Demographic and Clinical Characteristics

Between 1 October 2011 and 31 August 2018, 382 patients were included in the Uphold^TM^ group. Between 1 May 2019 and 31 December 2020, 84 patients were included in the ASSLF group (Table 1). A history of treatment for SUI was more frequent in the ASSLF group (13 out of 84 (15.9%) vs. 32 out of 382 (8.4%) in the Uphold^TM^ group; *p* = 0.04), as was the proportion of outpatient procedures (28 out of 84 (37.8%) vs. 66 out 382 (17.3%), respectively; *p* < 0.001). There were no other statistically significant intergroup differences.

### 3.2. Immediate and Early Postoperative Complications

The prevalence of acute urinary retention was significantly lower in the ASSLF group (3.6%) than in the Uphold^TM^ group (14.1%; *p* < 0.01), as was the de novo SUI rate (11.9% vs. 33.8%, respectively; *p* < 0.01) (Table 2). However, the rates of anterior or intermediate prolapse recurrence were significantly higher in the ASSLF group than in the Uphold^TM^ group (6.0% vs. 1.3%, respectively; *p* < 0.01).

### 3.3. Univariate Analysis

The intergroup difference in the overall failure rate was still significant in the immediate postoperative period (5 out of 84 (6.0%) in the ASSLF group vs. 5 out of 382 (1.3%) in the Uphold^TM^ group; *p* < 0.01) (Table 3).

The presence of grade ≥2 rectocele was a risk factor for failure of the procedure (6 out of 103 (5.8%) for grade ≥2 rectocele vs. 3 out of 289 (1.0%) otherwise; *p* < 0.01) (Table 3).

When considering the patients in the ASSLF group, 37 out of 83 (44.6%) underwent prolapse surgery, 9 (10.8%) underwent subvesical plication, 10 (12.0%) underwent subvesical “plastron” surgery (raising of an anterior vaginal flap attached to the bladder and which is then buried under the vaginal suture), and 18 (21.7%) underwent subvesical plication of Halban’s fascia with overlapping sutures. The performance of a concomitant anterior procedure during ASSLF or Uphold^TM^ placement was associated with a significantly lower risk of failure (5 out of 419 (1.2%), vs. 5 out of 47 (10.6%) without a concomitant procedure; *p* < 0.01).

With regard to ASSLF failures specifically (Table 4), performing a concomitant anterior procedure (0 failures out of 5 (0%), vs. 5 failures out of 5 (0%) without concomitant procedures; *p* = 0.06) and performing dual anchorage through the sacrospinous ligament (0 failures out of 5 (0%), vs. 5 out of 5 (100%) without dual anchorage, *p* = 0.07) were not significantly associated with risk of failure.

### 3.4. Multivariate Analysis

The results of the multivariate analysis showed that a concomitant anterior compartment procedure was predictive of fewer failures (odds ratio (OR) [95% confidence interval (CI) = 0.30 [0.11; 0.83]; Table 5). Moreover, the presence of grade ≥2 posterior compartment prolapse was predictive of early recurrence (OR [95%CI] = 3.6 [1.32; 9.74]).

## 4. Discussion

We observed a statistically significant difference in the overall recurrence rate during the immediate postoperative period (6.0% in the ASSLF group vs. 1.3% in the Uphold^TM^ group). However, all of the recurrences occurred at the start of the ASSLF period. Furthermore, we modified the ASSLF procedure during this period by adding dual anchorage of the suture in the ligament and/or by performing concomitant anterior colporrhaphy; thereafter, we did not observe any ASSLF failures. The absence of factors significantly associated with ASSLF failure in our study (e.g., the absence of dual anchorage or a concomitant anterior procedure) might be due to a lack of power. However, our failure rate was slightly lower than the literature values of 8 to 12% [17,18,19].

We did not observe any exposure in the ASSLF group, even though this type of complication can occur after the concomitant placement of a sub-urethral sling. It is noteworthy that the frequency of exposure in the Uphold^TM^ group (6 out of 374, 1.6%) was lower than the literature values for this type of prosthesis [20] and lower than the value found in Maher et al.’s meta-analysis (11.3%) for several types of prostheses [7].

Furthermore, we found that the acute urinary retention rate was lower in the ASSLF group (3.6%) than in the Uphold^TM^ group (14.1%); this was probably due to the absence of mesh. The value of 3.6% is lower than those found in the literature [19,21], although the definition of acute urinary retention tends to differ from one study to another. Mesh placement may result in anatomical changes in the urethra and bladder, and thus functional changes in the voiding mechanism [22]; these are thought to be less significant for native tissue repair. However, these results are difficult to interpret because (i) some of the patients in our study underwent urinary incontinence surgery concomitantly with the anterior prolapse procedure (which would substantially increase the risk of an obstruction), and (ii) some patients in the ASSLF group had to be catheterized for a long time because of injury to the bladder.

Indeed, we observed a higher frequency of bladder injury in the ASSLF group (6.0% patients in the ASSLF group vs. 0% in the Uphold^TM^ group). We cannot readily explain this difference; the dissection was the same in the two groups, as were the reported frequencies of pain and hemorrhage. One possible explanation is related to the level of surgical expertise. The surgical team comprised not only experienced practitioners, but also young residents; a lack of experience might be a factor, even though the procedure is supposedly easy to perform [23]. The literature data show that the frequency of bladder injury was higher for mesh procedures than for native tissue repair [21], although the literature values were lower than those observed in the present study [17].

De novo SUI was more frequent in the Uphold^TM^ group (33.8% vs. 11.0% in the ASSLF group). It should be noted that the two groups were dissimilar in terms of a history of surgery for SUI, with twice as many previous operations in the ASSLF group. We hypothesize that mesh placement tends to overcorrect the prolapse, causing additional force to be applied to the anterior wall and thus increasing the risk of SUI [9,24]. Alternatively, a concomitant anterior procedure (often colporrhaphy) might provide additional support for the neck of the bladder and the urethra, and thus reduce the likelihood of de novo SUI rate [20]. These results were similar to those in the latest meta-analysis by Maher et al., which found a relative risk of 0.67 of de novo SUI for surgery with native tissue repair vs. mesh placement [7].

A concomitant anterior procedure was associated with a significantly lower recurrence rate—perhaps by strengthening the anterior compartment. We noted a trend towards more frequent postoperative rectocele in the Uphold^TM^ group. In our multivariate analysis, the presence of grade ≥2 rectocele before surgery was a risk factor for early failure. The literature data indicate that a third of patients [25] will experience prolapse recurrence in a compartment other than that operated on. We hypothesize that (i) because mesh insertion limits the risk of anterior recurrence, an imbalance in the pelvis is created and the forces are transferred to the posterior compartment, and (ii) mesh insertion worsens the associated non-symptomatic prolapse that is not detected at the initial consultation. 

As ASSLF produces less stiffness than mesh, it might distribute the forces more evenly and maintain a degree of elasticity in the anterior compartment. These results are consistent with those of the meta-analysis by Maher et al., who found a risk of apical or posterior prolapse recurrence of 18% for mesh insertion and between 5% and 18% for native tissue repair [7].

The present study had a number of strengths, including a series of consecutive patients with the same indications treated by the same small team of surgeons. Furthermore, the general characteristics of the ASSLF and Uphold^TM^ groups (and particularly the prolapse grade) were similar. The study’s limitations included a comparison of ASSLF with a technique that is no longer used. However, mesh insertion was the reference technique until the prosthesis was withdrawn from the market. Secondly, there was a risk of indication bias, but this was mitigated by the fact that the same surgeon set all the surgical indications for Uphold^TM^ and then ASSLF. He did not modify his practice by referring some of the patients to laparoscopic sacropexy, for example. Thirdly, the fact that the postoperative outcomes were evaluated by the surgeon who prescribed the procedure might have introduced classification bias. Fourthly, we focused initially on pre-op and immediate or early post-op complications with a short follow-up because (i) a certain number of patients only came to the 2-month post-op consultation and not to the following ones, and were then lost to follow-up, and (ii) the end of data collection took place at the same time as the last inclusions, so to have sufficient numbers at 1 or 2 years it would have been necessary to wait for a longer period. Nevertheless, data collection is still ongoing in order to increase the size of the cohort and to provide results on medium- and long-term follow-up. Lastly, given the retrospective nature of the study and the current ban on the use of mesh outside clinical trials, we were not able to randomize the patients.

ASSLF with double anchorage of the suture through the ligament has not been previously described. This technique has fairly low short-term failure and complication rates. Although our short-term results are promising, medium- and long-term data on failure rates are now required. It would also be useful to assess the impact of ASSLF on the patient’s sex life, relative to the known effects of prostheses such as Uphold^TM^ [26].

Given the higher failure rate for surgery with native tissue repair, it is essential to continue to compare techniques with vs. without mesh. The recently modified ASSLF appears to provide better results than before. Depending on the medium- and long-term results, further developments of this technique could be envisaged.

## 5. Conclusions

ASSLF appears to be a reasonable alternative to transvaginal mesh insertion in the management of cystocele; the early complication rate is slightly lower, but the early failure rate is slightly higher.

## Figures and Tables

**Table 1:** Demographic and clinical characteristics of the study participants (N = 466).

Variable	Uphold^TM^ (*n* = 382)	ASSLF (*n* = 84)	*p*-Value
Age	69 [11]	69 [8]	0.80
BMI	25.3 [5]	25.0 [5.7]	0.96
Vaginal parity	2 [1]	2 [1]	0.56
Caesarian parity	0 [0]	0 [0]	1
Smoking	15 (4.1)	4 (4.6)	0.53
Menopausal status	235 (98.7)	79 (95.2)	0.08
History of hysterectomy	78 (20.5)	15 (18.1)	0.79
History of prolapse surgery	68 (17.9)	20 (23.8)	0.21
History of SUI surgery	32 (8.4)	13 (15.9)	0.04
Frequency of spinal anesthesia	83 (21.7)	12 (14.8)	0.26
Concomitant vaginal surgery	74 (19.4)	21 (25.6)	0.41
Concomitant posterior surgery	41 (10.7)	10 (11.9)	0.75
Anterior compartment prolapse			0.15
Grade 1	8 (2.1)	1 (1.2)	
Grade 2	52 (13.7)	17 (20.5)	
Grade 3	276 (72.8)	61 (73.5)	
Grade 4	43 (11.4)	4 (4.8)	
Apical compartment prolapse			0.07
Grade 0	25 (7.2)	1 (1.2)	
Grade 1	33 (9.5)	12 (14.5)	
Grade 2	162 (46.4)	44 (53.0)	
Grade 3	98 (28.1)	17 (20.5)	
Grade 4	31 (8.9)	9 (10.8)	
Posterior compartment prolapse			0.89
Grade 0	128 (41.3)	38 (46.3)	
Grade 1	98 (31.6)	25 (30.5)	
Grade 2	58 (18.7)	13 (15.9)	
Grade 3	17 (5.5)	3 (3.7)	
Grade 4	9 (2.9)	3 (3.7)	
Ambulatory surgery	66 (17.3)	28 (37.8)	<0.01

IQR: interquartile range. BMI: body mass index. SUI: stress urinary incontinence. The data are quoted as the median [interquartile range] or n/N (%).

**Table 2:** Immediate and early postoperative complications in the two treatment groups (N = 466).

	Uphold^TM^ (*n* = 382)	ASSLF (*n* = 84)	
	Number (%)	Number (%)	*p*-Value
Acute urinary retention	54 (14.1)	3 (3.6)	<0.01
Bleeding, hematoma	15 (3.9)	3 (3.6)	0.88
Pain	6 (1.6)	1 (1.2)	0.79
Anterior/middle compartment recurrence	5 (1.3)	5 (6.0)	<0.01
Posterior compartment recurrence	12 (3.1)	1 (1.2)	0.33
Infectious complications	3 (0.8)	1 (1.2)	0.72
Bladder injury	0	5 (6.0)	<0.01
Prosthesis exposure	6 (1.6)	0	
De novo SUI	129 (33.8)	10 (11.9)	<0.01

ASSLF: anterior sacrospinous ligament fixation; SUI: stress urinary incontinence.

**Table 3:** Factors associated with failure of the surgical procedure in a univariate analysis (N = 466).

				*p*
Total		10/466	(2.2)	
Failure of the procedure				<0.01
	ASSLF	5/84	(1.3)	
	Uphold^TM^	5/382	(6.0)	
Age				0.80
	Failure	68.5	[7]	
	No failure	69	[10]	
Vaginal parity				0.27
	Failure	2	[1]	
	No failure	2	[1]	
Smoking				0.36
	Yes	1/19	(5.3)	
	No	9/417	(2.2)	
Menopausal				0.01
	Yes	6/314	(1.9)	
	No	2/7	(28.6)	
History of hysterectomy				0.44
	No	7/370	(1.9)	
	Yes	3/93	(3.2)	
History of prolapse surgery				0.10
	Yes	4/88	(4.6)	
	No	6/377	(1.6)	
History of surgery for SUI				0.61
	Yes	0/45	(0)	
	No	10/418	(2.4)	
Anesthesia				0.47
	General	7/365	(1.9)	
	Spinal and then general	0/3	(0)	
	Spinal	3/95	(3.3)	
Concomitant vaginal surgery				0.15
	Yes	4/95	(4.2)	
	No	6/368	(1.6)	
Concomitant posterior compartment surgery				0.08
	Yes	3/51	(5.9)	
	No	7/415	(1.7)	
Grade ≥2 anterior compartment prolapse				0.18
	Yes	9/453	(2.0)	
	No	1/9	(11.1)	
Grade ≥2 apical compartment prolapse				0.38
	Yes	10/361	(2.8)	
	No	0/71	(0)	
Grade ≥2 posterior compartment prolapse				<0.01
	Yes	6/103	(5.8)	
	No	3/289	(1.0)	
Concomitant anterior compartment surgery				<0.01
	Yes	5/419	(1.2)	
	No	5/47	(10.6)	

The data are quoted as the median [interquartile range] or n/N (%). SUI: stress urinary incontinence; ASSLF: anterior sacrospinous ligament fixation.

**Table 4:** Factors associated with failure of the ASSLF procedure in a univariate analysis (N = 5).

		n/N	%	*p*
Concomitant anterior procedure for ASSLF				0.06
	Yes	0/5	0	
	No	5/5	100	
Dual anchorage for ASSLF				0.07
	Yes	0/5	0	
	No	5/5	100	

ASSLF: anterior sacrospinous ligament fixation.

**Table 5:** Multivariate analysis of the risk of failure for factors with *p* < 0.10 in the univariate analysis (N = 457).

Failure		OR	95%CI	* p * -Value
Concomitant anterior procedure				
	No	1 (ref)		
	Yes	0.30	[0.11; 0.83]	0.02
Grade ≥2 posterior compartment prolapse				
	No	1 (ref)		
	Yes	3.60	[1.32; 9.74]	0.01
Concomitant posterior procedure				
	No	1 (ref)		
	Yes	0.47	[0.11; 1.93]	0.29

Menopausal status was excluded, due to a high proportion of missing data. CI: confidence interval; OR: odds ratio.

## Data Availability

Data from the analysis are available upon request from the authors.

## References

[1] Barber M.D., Maher C. (2013). Epidemiology and outcome assessment of pelvic organ prolapse. Int. Urogynecol. J..

[2] Fialkow M.F., Newton K.M., Lentz G.M., Weiss N.S. (2008). Lifetime risk of surgical management for pelvic organ prolapse or urinary incontinence. Int. Urogynecol. J..

[3] Olsen A.L., Smith V.J., Bergstrom J.O., Colling J.C., Clark A.L. (1997). Epidemiology of surgically managed pelvic organ prolapse and urinary incontinence. Obstet. Gynecol..

[4] Liedl B., Goeschen K., Durner L. (2017). Current treatment of pelvic organ prolapse correlated with chronic pelvic pain, bladder and bowel dysfunction. Curr. Opin. Urol..

[5] Brincat C.A., Larson K.A., Fenner D.E. (2010). Anterior Vaginal Wall Prolapse: Assessment and Treatment. Clin. Obstet. Gynecol..

[6] Summers A., Winkel L.A., Hussain H.K., DeLancey J.O. (2006). The relationship between anterior and apical compartment support. Am. J. Obstet. Gynecol..

[7] Maher C., Feiner B., Baessler K., Christmann-Schmid C., Haya N., Brown J. (2016). Surgery for women with anterior compartment prolapse. Cochrane Database Syst. Rev..

[8] https://www.fda.gov/news-events/press-announcements/fda-takes-action-protect-womens-health-orders-manufacturers-surgical-mesh-intended-transvaginal.

[9] Daniel A., Tapio V., Ellström E.M., Susanne A., Christian F. (2011). Anterior Colporrhaphy versus Transvaginal Mesh for Pelvic-Organ Prolapse. N. Engl. J. Med..

[10] Amreich J. (1950). Technic in vaginal surgery. Arch. Gynakol..

[11] Richter K. (1968). The surgical anatomy of the vaginaefixatio sacrospinalis vaginalis. A contribution to the surgical treatment of vaginal blind pouch prolapse. Geburtshilfe Frauenheilkd..

[12] Solomon E.R., St Marie P., Jones K.A., Harmanli O. (2020). Anterior Bilateral Sacrospinous Ligament Fixation: A Safe Route for Apical Repair. Urogynecology.

[13] Winkler H.A., Tomeszko J.E., Sand P.K. (2000). Anterior sacrospinous vaginal vault suspension for prolapse. Obstet. Gynecol..

[14] Goldberg R.P., Tomezsko J.E., Winkler H.A., Koduri S., Culligan P.J., Sand P.K. (2001). Anterior or posterior sacrospinous vaginal vault suspension: Long-term anatomic and functional evaluation. Obstet. Gynecol..

[15] Renard N., Bartolo S., Giraudet G., Declas E., Rubod C., Cosson M. (2020). Feasibility of vaginal mesh for anterior vaginal wall prolapse in an ambulatory setting: A retrospective case series. J. Gynecol. Obstet. Hum. Reprod..

[16] Barber M.D., Brubaker L., Nygaard I., Wheeler T.L., Schaffer J., Chen Z., Spino C. (2009). Defining Success After Surgery for Pelvic Organ Prolapse. Obstet. Gynecol..

[17] Plair A., Dutta R., Overholt T.L., Matthews C. (2021). Short-term outcomes of sacrospinous hysteropexy through an anterior approach. Int. Urogynecol. J..

[18] Cespedes R. (2000). Anterior approach bilateral sacrospinous ligament fixation for vaginal vault prolapse. Urology.

[19] Siddiqui S., Gayen A., Wong V. (2021). Short-term outcomes of anterior approach sacrospinous ligament fixation for apical vaginal prolapse—A retrospective study. Facts, Views Vis. ObGyn.

[20] Rahkola-Soisalo P., Mikkola T.S., Altman D., Falconer C. (2019). Pelvic Organ Prolapse Repair Using the Uphold Vaginal Support System: 5-Year Follow-Up. Urogynecology.

[21] Delacroix C., Allegre L., Chatziioannidou K., Gérard A., Fatton B., de Tayrac R. (2022). Anterior bilateral sacrospinous ligament fixation with concomitant anterior native tissue repair: A pilot study. Int. Urogynecol. J..

[22] Altman D., Mikkola T.S., Bek K.M., Rahkola-Soisalo P., Gunnarsson J., Engh M.E., Falconer C., For the Nordic TVM group (2016). Pelvic organ prolapse repair using the Uphold^TM^ Vaginal Support System: A 1-year multicenter study. Int. Urogynecol. J..

[23] Petruzzelli P., Chiadò Fiorio Tin M., Cosma S., Parisi S., Garofalo A., Todros T. (2016). Combined sacrospinous hysteropexy and cystopexy using a single anterior incision. Int. J. Gynecol. Obstet..

[24] Alas A.N., Chinthakanan O., Espaillat L., Plowright L., Davila G.W., Aguilar V.C. (2017). De novo stress urinary incontinence after pelvic organ prolapse surgery in women without occult incontinence. Int. Urogynecol. J..

[25] Clark A.L., Gregory T., Smith V.J., Edwards R. (2003). Epidemiologic evaluation of reoperation for surgically treated pelvic organ prolapse and urinary incontinence. Am. J. Obstet. Gynecol..

[26] Rahkola-Soisalo P., Altman D., Falconer C., Morcos E., Rudnicki M., Mikkola T.S. (2017). Quality of life after Uphold^TM^ Vaginal Support System surgery for apical pelvic organ prolapse—A prospective multicenter study. Eur. J. Obstet. Gynecol. Reprod. Biol..

